# Extrapyramidal symptoms as a consequence of organophosphate poisoning: Insights from a clinical case

**DOI:** 10.1192/j.eurpsy.2021.2063

**Published:** 2021-08-13

**Authors:** J. Martins Correia, S. Freitas Ramos, M.I. Fonseca Marinho Vaz Soares, B. Jesus, D. Cruz E Sousa, S. Caetano

**Affiliations:** Department Of Psychiatry And Mental Health, Local Health Unit of Guarda, Guarda, Portugal

**Keywords:** organophosphate poisoning, extrapyramidal symptoms, extrapyramidal syndrome, Catatonia

## Abstract

**Introduction:**

The development of an extrapyramidal syndrome (EPS) is assumed to be a potential and important consequence of organophosphate poisoning (OP). Even though its causal relationship is firmly established, the information available in the literature regarding the orientation to be given is scarce, and its approach remains shrouded in a significant degree of uncertainty. Catatonia, as a neuropsychiatric condition, may present a marked overlap with the set of extrapyramidal symptoms developed after OP. Does the overlap between the symptoms seen in catatonia and in EPS make differential diagnosis fundamental or does it have no relevance in relation to the approach to be established?

**Objectives:**

To discuss the therapeutic approach to be implemented in the extrapyramidal symptoms resulting from OP and reflect on the overlap between catatonia and EPS.

**Methods:**

Presentation of a clinical case and review of the literature.

**Results:**

A 50-year-old woman with major depressive disorder developed a condition marked by exuberant extrapyramidal symptoms 3 weeks after OP. Significant stiffness, tremor, dysphagia and facial hypomimia were some of the symptoms observed. Therapy was started with amantadine 100mg daily, with complete resolution of the symptoms after 5 days. Follow-up revealed reversal of extrapyramidal symptoms, in the absence of any neuroimaging changes or any other neuropsychiatric manifestations.

**Conclusions:**

The possible overlap between catatonia and EPS is remarkable. The two conditions, regardless of their differentiation, may benefit from an identical approach using dopaminergic drugs. The use of amantadine, even in low doses, may be an option in the rapid reversal of extrapyramidal symptoms resulting from OP.

**Disclosure:**

No significant relationships.

